# A Collaborative and Scalable Geospatial Data Set for Arctic Retrogressive Thaw Slumps with Data Standards

**DOI:** 10.1038/s41597-025-04372-7

**Published:** 2025-01-06

**Authors:** Yili Yang, Heidi Rodenhizer, Brendan M. Rogers, Jacqueline Dean, Ridhima Singh, Tiffany Windholz, Amanda Poston, Stefano Potter, Scott Zolkos, Greg Fiske, Jennifer Watts, Lingcao Huang, Chandi Witharana, Ingmar Nitze, Nina Nesterova, Sophia Barth, Guido Grosse, Trevor Lantz, Alexandra Runge, Luigi Lombardo, Ionut Cristi Nicu, Lena Rubensdotter, Eirini Makopoulou, Susan Natali

**Affiliations:** 1 Woodwell Climate Research Center, 149 Woods Hole Road, Falmouth, MA 02540-1644 USA; 2 Institute of Space and Earth Information Science, The Chinese University of Hong Kong, Shatin, N.T., Hong Kong, China; 3 Department of Natural Resources and the Environment, University of Connecticut, Storrs, CT 06269 USA; 4 Alfred Wegener Institute, Permafrost Research Section, Telegrafenberg A45, 14473 Potsdam, Germany; 5 School of Environmental Studies, University of Victoria David Turpin Building, B243 Victoria, BC Canada; 6 Helmholtz Centre Potsdam GFZ German Research Centre for Geosciences, Telegrafenberg, 14473 Potsdam, Germany; 7 Faculty of Geo-Information Science and Earth Observation, University of Twente, Enschede, the Netherlands; 8 High North Department, Norwegian Institute for Cultural Heritage Research (NIKU), Fram Centre, N-9296 Tromsø, Norway; 9 Geohazard and Earth Observation, Geological Survey of Norway (NGU), P.O. Box 6315 Torgarden, 7491 Trondheim, Norway; 10 Department of Arctic Geology, The University Centre in Svalbard (UNIS), P.O. Box 156, 9171 Longyearbyen, Norway; 11 Geography Research Unit, University of Oulu, Oulu, 90014 Finland

**Keywords:** Cryospheric science, Environmental impact

## Abstract

Arctic permafrost is undergoing rapid changes due to climate warming in high latitudes. Retrogressive thaw slumps (RTS) are one of the most abrupt and impactful thermal-denudation events that change Arctic landscapes and accelerate carbon feedbacks. Their spatial distribution remains poorly characterised due to time-intensive conventional mapping methods. While numerous RTS studies have published standalone digitisation datasets, the lack of a centralised, unified database has limited their utilisation, affecting the scale of RTS studies and the generalisation ability of deep learning models. To address this, we established the Arctic Retrogressive Thaw Slumps (ARTS) dataset containing 23,529 RTS-present and 20,434 RTS-absent digitisations from 20 standalone datasets. We also proposed a Data Curation Framework as a working standard for RTS digitisations. This dataset is designed to be comprehensive, accessible, contributable, and adaptable for various RTS-related studies. This dataset and its accompanying curation framework establish a foundation for enhanced collaboration in RTS research, facilitating standardised data sharing and comprehensive analyses across the Arctic permafrost research community.

## Background & Summary

The terrestrial permafrost region in the northern hemisphere stores roughly 1600 Pg of carbon, and permafrost is warming and thawing, both through more gradual active layer deepening and through relatively abrupt thermokarst processes^1–3^. Recent climate change has decreased permafrost extent by approximately 7%^4^, and losses of up to 70% are expected by 2100 under low mitigation scenarios^5^. Permafrost thaw can result in rapid decomposition of soil carbon, releasing greenhouse gasses (GHG, mainly CO_2_ and CH_4_) into the atmosphere^2,6^. However, the minority of current Earth system models that include permafrost carbon only represent ‘gradual thaw’^7^, neglecting the impact of rapid terrain collapse following thaw (i.e., thermokarst) and its biogeochemical consequences. Early predictions of future permafrost carbon release estimated a significant increase in GHG emissions attributed to intensifying thermokarst activity^6^. One fundamental limitation for understanding the importance of abrupt thaw on Arctic landscapes and carbon feedback is the lack of geospatial products describing abrupt thaw distribution and changes over time.

Retrogressive Thaw Slumps (RTS) are slope failures that develop in ice-rich terrain. They are one of the most rapid thermokarst features^8^ in permafrost regions. RTS typically feature a steep back wall and a low-angle bottom with displaced soil. Often initiated near water bodies, the slump deposit is rapidly removed, leaving a persistent scar. The process may continue as the active layer develops, causing back-scarp instability. RTS expansion through headwall retreat can persist until substrate or thermal conditions change, or when displaced vegetation insulates the ice-rich scarp from further thawing^9,10^. Well-developed RTS scars or depletion areas can be easily recognised in the landscape because they commonly appear as a horseshoe-shaped depression with a different surface pattern than the surroundings, often connected at one end to a river, a thermokarst lake, or shoreline^11^. Individual RTS typically occur over relatively small areas (<40 ha) at the landscape scale, yet can mobilise permafrost substrate and affect infrastructure, such as buildings, roads, railways, and airports^12,13^, industrial sites^14^, ecosystems^15–17^, and biogeochemical processes across watershed scales^18–21^; at the same time they can pose a serious threat to the sustainable development and resilience of Arctic communities^22^ and cultural heritage sites^23,24^. While rising air temperatures and increasing precipitation at northern high latitudes^25–27^ are expected to increase RTS abundance and activity^28,29^, understanding of potential ecological and societal impacts across the permafrost region is hindered by challenges in broadly identifying and mapping RTS features. RTS have been studied using observational methods ranging from satellite remote sensing to airborne photogrammetry and field observations. Nevertheless, the spatial distribution and temporal progression of RTS features across large scales are not well characterised^30,31^.

Among all the types of RTS studies, deep learning (DL) studies are the most data-intensive ones due to the state-of-the-art training paradigm of approximating a black-box function using extensive amounts of data. In the past three years, DL methods, especially convolutional neural networks (CNNs) and vision transformers (ViTs), have been increasingly used to detect RTS features in combination with satellite remote sensing^31–35^. These recent DL model applications are largely limited to a few regions where RTS features have been sampled and manually delineated. So far no model can detect and map RTS across the Arctic. One reason is that RTS’s geomorphic and land cover characteristics vary widely across permafrost regions, and non-RTS background features can closely resemble RTS features^34^. This variability is a function of interrelated geomorphic, geological and ecological processes such as degree of RTS activity and vegetation growth^36,37^, terrain characteristics (e.g. slope, ice content), and age since RTS initiation. Therefore, the sampling of training data needs to reflect the full spectrum of RTS appearance across the Arctic, rather than relying on RTS features from a few local regions to represent RTS across the circumpolar domain. This is particularly important because DL methods rely heavily on the amount and representativeness of the training data to achieve high accuracy and generalisation across regions. Unfortunately, RTS training data acquisition is costly in terms of time and resources, making it challenging for individual lab groups to compile a robust training dataset for broader mapping efforts. However, these challenges can largely be addressed with collaboration and thoughtful data sharing across lab groups, enabling the creation of a pan-Arctic RTS training set to build a DL model for pan-Arctic RTS detection.

While developing robust training datasets is essential for improving DL model performance, an often overlooked yet crucial aspect is the proper representation of landscapes without RTS features. For DL models to accurately identify RTS, they must learn from both RTS features (positive data) and verified RTS-absent areas (negative data). This comprehensive approach serves two purposes: enabling systematic geographical comparisons of RTS distribution and providing balanced training data for DL models. A comprehensive training dataset must capture the complete data distribution, encompassing both positive samples that represent the full spectrum of RTS morphological characteristics and negative samples that effectively characterise the diverse non-RTS landscapes across the permafrost region. This balanced representation is particularly critical because RTS features occupy only a small fraction of the Arctic landscape, creating a significant class imbalance challenge. When trained solely on positive data, models incorrectly learn that RTS are common features, leading to high false-positive rates during prediction^38^. While computational solutions exist, such as adjusting sample weights and loss functions, the fundamental solution lies in systematically sampling and verifying negative data during the training data development phase. Previous studies^31,34^ have demonstrated this approach regionally but pan-Arctic negative data is still needed for a pan-Arctic RTS model. Beyond DL applications, negative data also plays a vital role in RTS susceptibility studies, as demonstrated by Makopoulou *et al*.^39^ and Luo *et al*.^40^ in their machine learning-based assessments of RTS susceptibility across the northern hemisphere.

To solve the challenges discussed above, we developed a comprehensive data set - the Arctic Retrogressive Thaw Slumps (ARTS) - including 20 data sets (Table 1) for RTS digitisation polygons as ‘positive data’ or verified RTS-absent regions as ‘negative data’. Each source data set has a few hundred to several thousand manually digitised RTS instances. These data sets are manually digitised and verified by permafrost domain experts and collectively cover a wide spectrum of Arctic RTS environments. The regions included the most-studied Arctic RTS hotspots such as Siberia, the Canadian Arctic Archipelago, the Svalbard Archipelago, and the Yukon Territory and Northwest Territories (NWT). While the ARTS data set is built for all ranges of RTS studies, we set the standard of the ARTS to fulfil the highest demand of large-scale DL modelling, including computer vision and time-series forecasting. The ARTS dataset is designed around three core principles: scalability, interoperability, and informativeness. Scalability ensures the database can efficiently update fast-changing RTS time series, incorporate new entries, and manage data growth, allowing it to evolve with ongoing research. Interoperability is achieved through unified metadata and data format standards, implementing an indexing system using a Unique Identifier (UID) generation algorithm, and features that facilitate seamless collaboration and easy access for contributors and users alike. Informativeness is maintained by mandating key metadata for reproducibility, including both RTS-present and RTS-absent digitisations, and utilizing peer-reviewed data sources. These principles collectively ensure that ARTS remains a dynamic, accessible, and reliable resource for comprehensive Arctic permafrost research, capable of adapting to the evolving needs of the scientific community.

**Table 1:** Summary of RTS data sources.

Source	Verified RTS feature	Verified non- RTS feature	Type	Regions
Nitze *et al*.^31^ (Ver.2)	3,579	1,300	polygon	25 locations in Russia, Canada and Alaska
Yang *et al*.^34^	855	3,218	polygon	Yamal-Gydan, Banks Island, Herschel Island, Horton Delta, Kolguev Island, Lena River, Tuktoyaktuk Pen.
Huang *et al*.^32^	621		polygon	Willow River, Hot Weather Creek, Banks Island
Witharana *et al*.^33^	356		polygon	Eureka Sound Lowlands
Lantz *et al*.^48^	669		polygon	Eastern Banks Island
Huang *et al*.^49^	2,494		bounding box	Pan-Arctic
Bernhard *et al*.^50^	1,832		point	Peel, Banks, Tuktoyaktuk, Ellesmere, Noatak, Yamal, Gydan, Taymyr Chukotka
van der Sluijs *et al*.^41^	2,660		polygon	Peel Plateau and Anderson Plain, Tuktoyaktuk Coastlands, Northwest Territories
Bernhard *et al*.^51^	1,487		polygon	Northern Taymyr Peninsula in Siberia, Russia
Lin *et al*.^52^	365		polygon	Banks Island and Victoria Island, Canada
Ramage *et al*.^53^	286		polygon	Yukon Coast, Canada
Nicu *et al*.^10^	562		polygon	Nordenskiöld Land, Svalbard, Norway
Elia *et al*., in prep.	690		polygon	Andrée and Dickson Land areas, northern Svalbard, Norway
Leibman *et al*.^43^	97		polygon	Yamal-Gydan, Russia
Barth *et al*.^54^	3,461		polygon	Novaya Zemlya Archipelago, Kolguev Island, Bol’shoy Lyakhovsky Island, and Taymyr Peninsula
Runge *et al*.^55^	1,769		polygon	Chukotka Coast, Iultinsky, Lower Lena, West Taymyr, Chokurdakh
Zwieback *et al*.^56^	165		polygon	Tuktoyaktuk, the Lena River delta area
Swanson *et al*.^57^	1309		polygon	Northern Alaska
Noerling *et al*.^58^	87		polygon	Yamal Peninsula
Makopoulou *et al*.^39^		15,905	polygon	Pan-Arctic
Total RTS digitisations count	23,529	20,434		

We designed a Data Curation Framework to establish a standardised, scalable, and collaborative approach to managing geospatial RTS data across the Arctic. Its primary intention is to overcome challenges associated with compiling diverse datasets from multiple sources, ensuring consistency, and facilitating ongoing contributions. The framework encompasses a unified metadata structure, a robust unique identifier system, standardised naming conventions and units, protocols for handling intersecting digitisations, and data storage and contribution guidelines. By implementing these standards, the framework aims to enhance data quality, improve interoperability, and streamline the process of integrating new information. This approach not only ensures the long-term usability and reliability of the ARTS dataset but also promotes collaboration among researchers, ultimately advancing our understanding of Arctic permafrost dynamics and their global implications. The Framework is not only a design but also a mature implementation, we provided tested codes and tutorials in Python and R for automated processing and streamlining the metadata formatting process in the ARTS GitHub repository (https://github.com/whrc/ARTS).

## Methods

### Data Curation Framework

The Data Curation Framework is our proposed standard and guideline for RTS data set creation, compilation, storage and future contribution. It also regulates the metadata requirements, formatting and indexing. The processing and compilation of the component raw data sets of the ARTS strictly followed the guidance of the Framework. Individual components of the Framework for RTS data curation are described in the following subsections.

#### Collating Standalone Data Sets

We compiled 20 peer-reviewed RTS digitisation datasets (Table 1), obtained through published links or directly from corresponding authors. The data collating process involved three main steps guided by the Framework. First, we cleaned the data by verifying and eliminating redundancies, while supplementing missing metadata using published information and direct communication with source laboratories. Next, we standardised the datasets by harmonising metadata field names, formats, and units. We generated UIDs for each polygon, assigning identical UIDs to multiple delineations of the same RTS instance due to temporal changes or different imagery sources. Relationships between intersecting digitisations were documented. Finally, we integrated all processed datasets into a comprehensive GeoJSON file. This rigorous approach ensures a consistent, high-quality compilation of RTS data suitable for large-scale analysis and future contributions. All raw RTS data sets that were collated for the ARTS can be downloaded from the GitHub repository (https://github.com/whrc/ARTS/tree/main/raw_data).

#### Metadata Formatting

Merging the metadata of the raw data sets presented challenges including inconsistent units, repeated or redundant IDs, and inconsistent or ambiguous attribute names. Here we present a standard metadata system that regulates critical information for each RTS digitisation (Table 2). This will facilitate creating, merging, and utilising future data sets. The first nine columns (Required-True) are mandatory attributes used for UID generation that provide essential metadata with a fixed entry format and order. The required metadata will be used as inputs for the data compilation code to produce auto-generated metadata. Creators can customise extra columns to add task-specific information after the auto-generated columns, for example, whether field verification has been conducted. The default entry for not-applicable optional metadata is *null*.

**Table 2:** Metadata Format Summary.

FieldName	Format	Required	Description
CentroidLat	Decimal Degrees	True	Polygon centroid latitude in EPSG:4326, round off to 5 decimal places
CentroidLon	Decimal Degrees	True	Polygon centroid longitude in EPSG:4326, round off to 5 decimal places
RegionName	String	True	Name of the geographical region
CreatorLab	String	True	Data creator and associated organization
BaseMapDate	String	True	Date of base map used for RTS delineation in YYYY-MM-DD for a single date, range of dates should be separated by a comma
BaseMapSource	String	True	Name of the satellite sensor used for RTS digitisation
BaseMapResolution	Number	True	Resolution of the imagery used for RTS digitisation (meters)
TrainClass	String	True	‘Positive’ for genuine RTS and ‘Negative’ for background
LabelType	String	True	Type of digitisation, e.g. ‘Polygon’, ‘BoundingBox’
MergedRTS	String	Auto-Generated	UIDs of intersecting RTS that merged into one RTS
SplitRTS	String	Auto-Generated	UID of RTS that split into multiple RTS
NewRTS	String	Auto-Generated	UIDs of intersecting RTS that formed on top of a stabilized RTS scar
StabilizedRTS	String	Auto-Generated	UIDs of intersecting stabilized RTS scars
UnknownRelationship	String	Auto-Generated	UIDs of intersecting RTS with unknown relationship
ContributionDate	String	Auto-Generated	Date of contribution to the ARTS main file in YYYY-MM-DD
UID	36-character alphanumeric string	Auto-Generated	Unique identifier generated using uuid5 by concatenating all ‘Required-True’ fields as a single string
CustomColumn1 (e.g. Area)	Custom (e.g. Number)	Optional	e.g. Area of the RTS polygon in km^2^
CustomColumn2 (e.g. State)	Custom (e.g. String)	Optional	e.g. Active state of the RTS polygon

In Table 2, the first column ‘FieldName’ lists the unified metadata names in the ARTS. The following columns ‘Format’, ‘Required’ and ‘Description’ are detailed standards and text descriptions that define the metadata. ‘CentroidLat’ and ‘CentroidLon’ are the latitude and longitude of the RTS centroid in decimal degrees, rounding off to 5 decimal places provides < 1m accuracy which is adequate for RTS features. This is useful for spatial distribution analysis or filtering based on coordinates. ‘RegionName’ is useful when filtering based on pre-defined regions or comparing regions. ‘CreatorLab’ records the data creator and affiliated organisation. This is useful when filtering based on creator or institution, and to credit the creator. ‘BaseMapDate’, ‘BaseMapSource’ and ‘BaseMapResolution’ record critical information about the base map used to create the polygons. This is useful for adapting polygons to another base map source. ‘TrainClass’ indicates if the data entry is a genuine RTS polygon or a non-RTS background tile, which is essential for providing both positive and negative examples to train deep learning models. ‘LabelType’ indicates the type of RTS digitisation, commonly provided as polygons (vectors) but could also be provided as bounding boxes or points. ‘MergedRTS’ and ‘StabilisedRTS’ are columns used to link the replacement relationship of new and old RTS.

Finally, all 20 data sets originally adopted sequential numbers to index the polygons. Ordinal number indices are ideal for small and simple data sets. However, for a large data set that expects frequent editing, updating and merging operations, numeric indices bear problems related to repetition, insertion and deletion at a massive scale. Therefore, a more systematic and robust indexing system is needed. This system should be 1) immune to adding, deleting, shuffling, or mixing, 2) reproducible, 3) unique to each RTS instance and consistent through time, and 4) easy to generate and maintain. We therefore utilised, and propose for future use, the Unique Identifier (UID) system which generates a unique 36-character alphanumeric ID string from a given input. The RTS UID is generated from a seeded random generator process, and the likelihood of repeated UID is extremely small and can be safely ignored. This guarantees unique IDs can be generated by different organisations without communication. To achieve reproducibility, all ‘Required=True’ metadata entries should be concatenated to a single string to be used as a generation seed. To ensure that the same RTS instance has the same UID across polygons that were delineated at different points in time, we require generating a UID for the earliest polygon and applying this UID to all subsequent polygons. Recognising the possibility that new contributions to the dataset could include polygons that correspond to features that already exist in the dataset, we will require that all new contributions be checked for intersections with the current version of the dataset, and the UID of any features which already exist in the dataset will be used.

RTS features exhibit complex spatial and temporal relationships, as new RTS can develop within stabilised scars and adjacent RTS may merge over time^41–43^. These dynamics necessitate a robust system for tracking RTS relationships in the geospatial database^44^. The UIDs for intersected RTS instances will be categorised by their relation. For example, in cases where multiple RTS features merge into one, the merged feature should receive a new UID, and the UIDs of the component features should be included in the ‘MergedRTS column’. Additionally, a newly initiated RTS feature on an old, stabilised scar should receive a new UID, and the UID of the old RTS should be included in the ‘StabilisedRTS’ column. To make this process easy for contributors, we have created publicly available automation scripts in Python and R to ensure that the UIDs meet all requirements and that the metadata formatting is correct. In total, there are five different categories of RTS relations in the ARTS, see Table 2. Using RTS IDs and required metadata columns, it will be possible to easily differentiate 1) the digitisation of the same RTS by multiple groups using different base maps, 2) the digitisation of the same RTS instance at different times, 3) the digitisation of a new RTS which initiated on an older, stabilised RTS, and 4) the digitisation of a coalesced RTS which formed when two adjacent RTS merged.

#### Intersecting digitisations and UID generation rule

Intersecting digitisations are due to mainly three reasons: 1) multiple groups digitising the same RTS with different definitions or standards, 2) a series of digitisations of RTS development through time, i.e. a time-series, and 3) digitisation on images acquired on different dates, mosaics or different sensors.

All new entries need to be checked for repeat delineations of the same RTS feature. For this purpose, we have published scripts on GitHub (https://github.com/whrc/ARTS) in Python and R that check all new RTS polygons for intersections with polygons in the published RTS data set. In brief, the scripts allow contributors to easily create a GeoJSON that enumerates all overlapping polygons between the new and published data sets. This file can then be visualised over imagery so that the contributor can determine whether polygons with intersections in the published data set are new RTS features or repeat delineations of previously observed RTS features. Using this information, UIDs can then be assigned to new RTS features, while repeat RTS features inherit the UID of the previously delineated polygon. In cases where a new RTS feature has initiated on top of an old, stabilised RTS scar or multiple RTS features have merged, the polygon is considered a new RTS feature for UID generation purposes.

#### Naming Custom Columns

The full names of custom columns should be named using Pascalcase: complete words delimited by capital letters. This is called the FieldName, which lists the column names presented in the data set (e.g. Table 3). Before adding a new custom column, contributors need to check with the late.

**Table 3:** Example of valid FieldName and Values for mandatory and optional columns.

FieldName	Value
CentroidLat	−77.508333
CentroidLon	164.754167
RegionName	‘Yamal-Gydan, NW Russia’
CreatorLab	‘JDean, Woodwell’
BaseMapDate	‘2020-12-31,2022-11-29’
BaseMapSource	‘WorldView-2’
BaseMapResolution	4
TrainClass	‘Positive’
LabelType	‘Polygon’
MergedRTS	‘a6cf10e7-6515-5ab7-b7bb-66606b16ef10’, ‘dd137c86-6db6-57da-bb46-961c89845a97’
StabilizedRTS	null
ContributionDate	‘2023-12-31’
UID	‘acde070d-8c4c-4f0d-9d8a-162843c10333’
Area	10
State	‘Active’

#### Units

In the Metadata Format Summary (Table 2) Description column, we specified the type of default International System of Units (SI units) that should be used for scalar metadata, therefore unit should not be repeatedly specified in the metadata entries. For a new custom column without an existing description in the Metadata Format Summary, a suitable SI unit should be specified and consistently used for future entries. For instance, CustomColumn for RTS areas is defined in Table 2 row 14 and specified its unit in km^2^, therefore the unit is omitted in the actual metadata entry (Table 3 row 14).

#### RTS-present and RTS-absent data

**Fig. 1:**
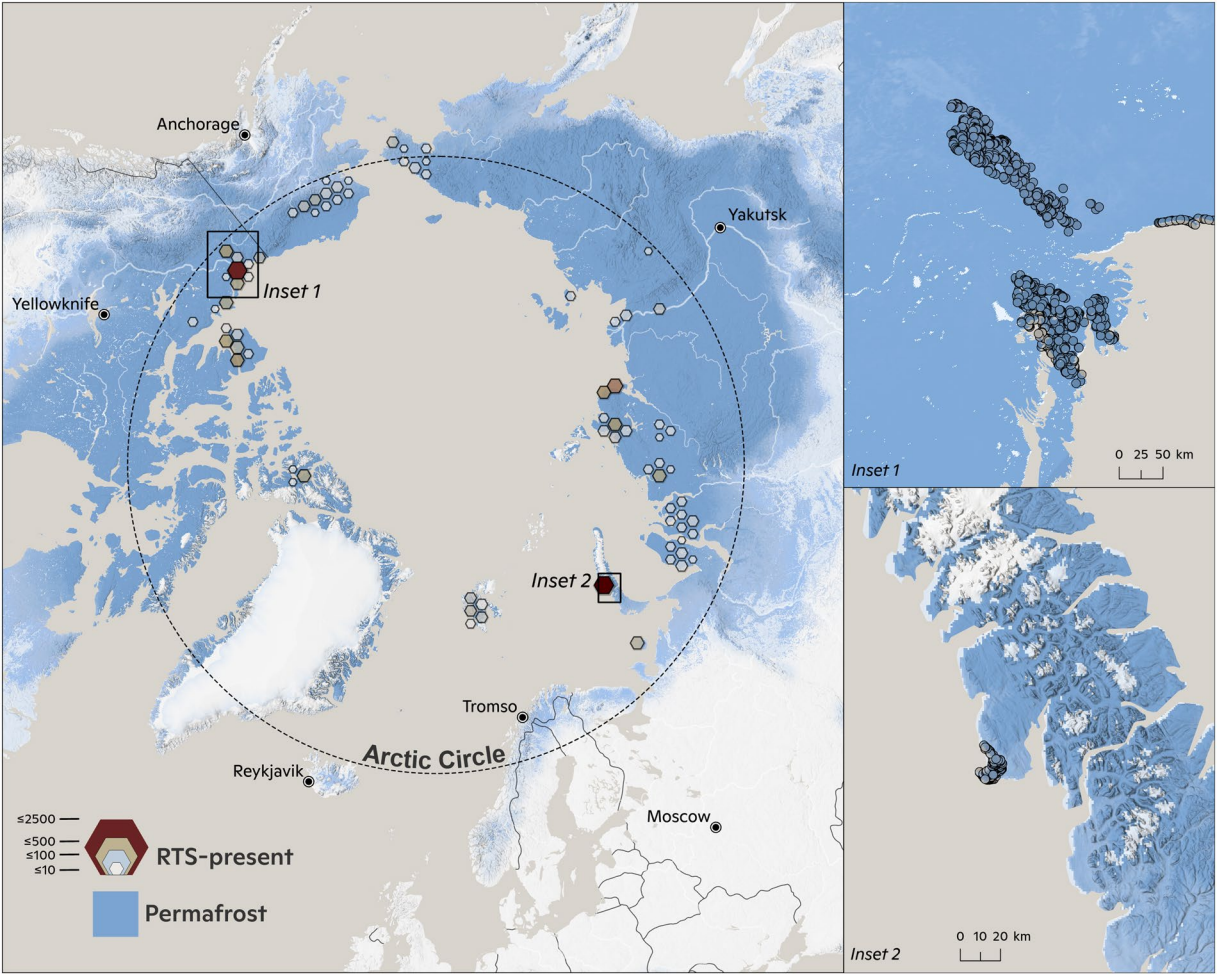
Visualisation of the distribution of RTS-present digitisations in the ARTS data set. Inset 1,2 shows the highly heterogeneous RTS density distribution where RTS occurrence tend to be hihgly clustered in hot-spot regions.

**Fig. 2:**
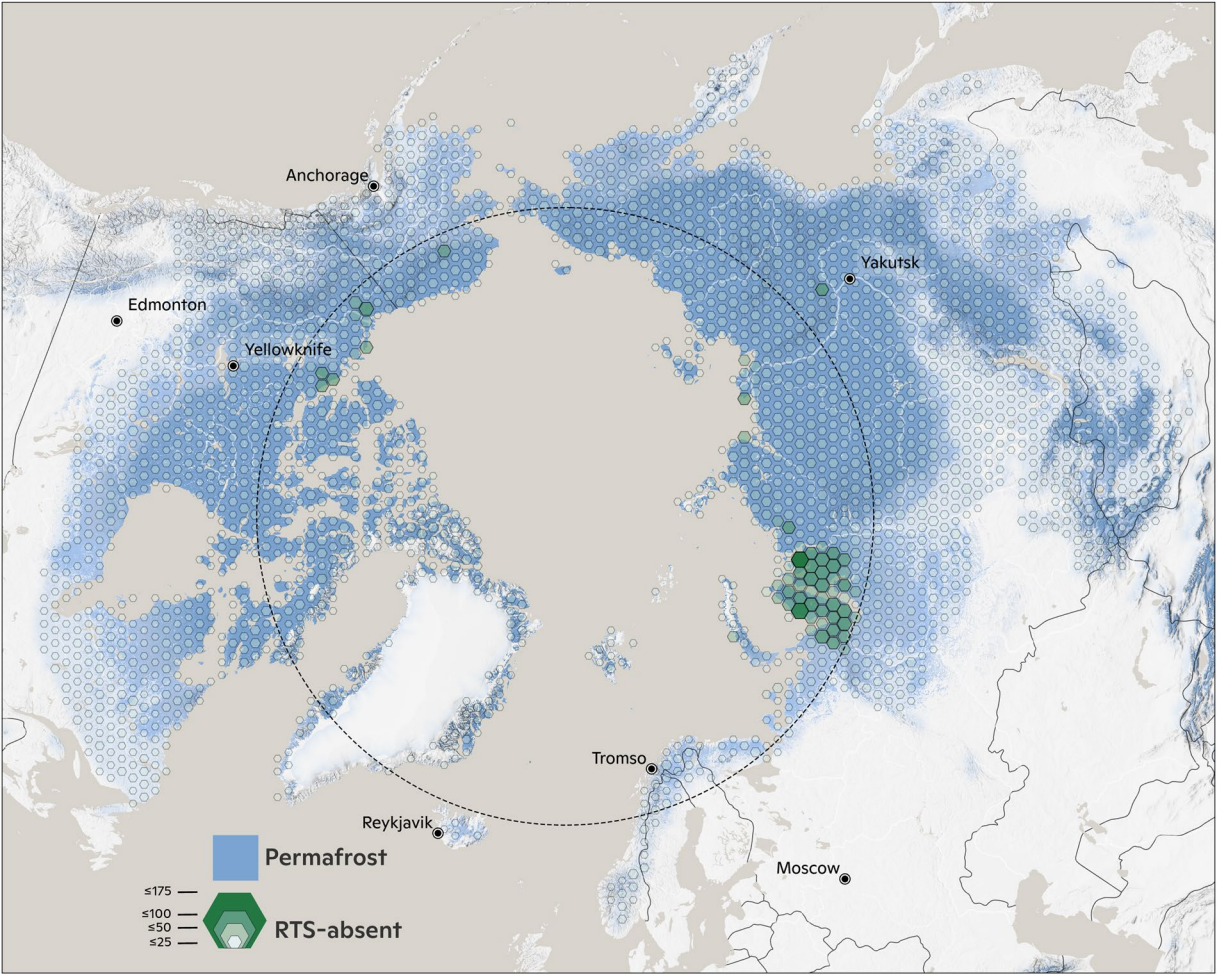
Visualisation of the distribution of RTS-absent digitisations in the ARTS data set. The RTS absent data is relatively homogeneously sampled across the Arctic permafrost regions compared with the RTS-present data.

The ARTS data set collected 23,529 manually digitised RTS-present polygons (Fig. 1), as well as 20,434 RTS-absent digitisations (Fig. 2), sampled across the whole Arctic region. RTS-present (positive) and RTS-absent (negative) data are verified and indexed with UIDs. Positive data should ideally be added as polygons, but other digitisation types such as bounding boxes or points are also accepted. Verified negative data should be added as bounding boxes or polygons excluding any genuine RTS.

#### Data Storage Format

Most of the original data sets were provided as Esri shapefiles - a vector data storage format that stores the location, shape and custom attributes of geographic features, only one data set is in the format of Geopackage and two in .tab files. Although shapefile has been the most widely used format for geographic vector data, it suffers from several limitations, such as being slow, restricted lengths for column names, and being composed of multiple files, which can result in version control and file sharing problems. We chose GeoJSON as the data storage format because it is an open standard vector geospatial data format that is widely used and easily integrated with many desktop geospatial packages and web platforms such as QGIS, ArcGIS, and Google Earth Engine. Additionally, raw GeoJSON is directly readable and editable by humans, making it easy to inspect and maintain. Our team also considered using the GeoPackage format but ultimately rejected it mainly because of its relatively lower adoption rate and higher complexity.

## Data Records

The ARTS data set v.1.0.0 is available at the Arctic Data Center^45^ (arcticdata.io/catalog/view/doi:10.18739/A2PK0738B).

## Technical Validation

**Fig. 3:**
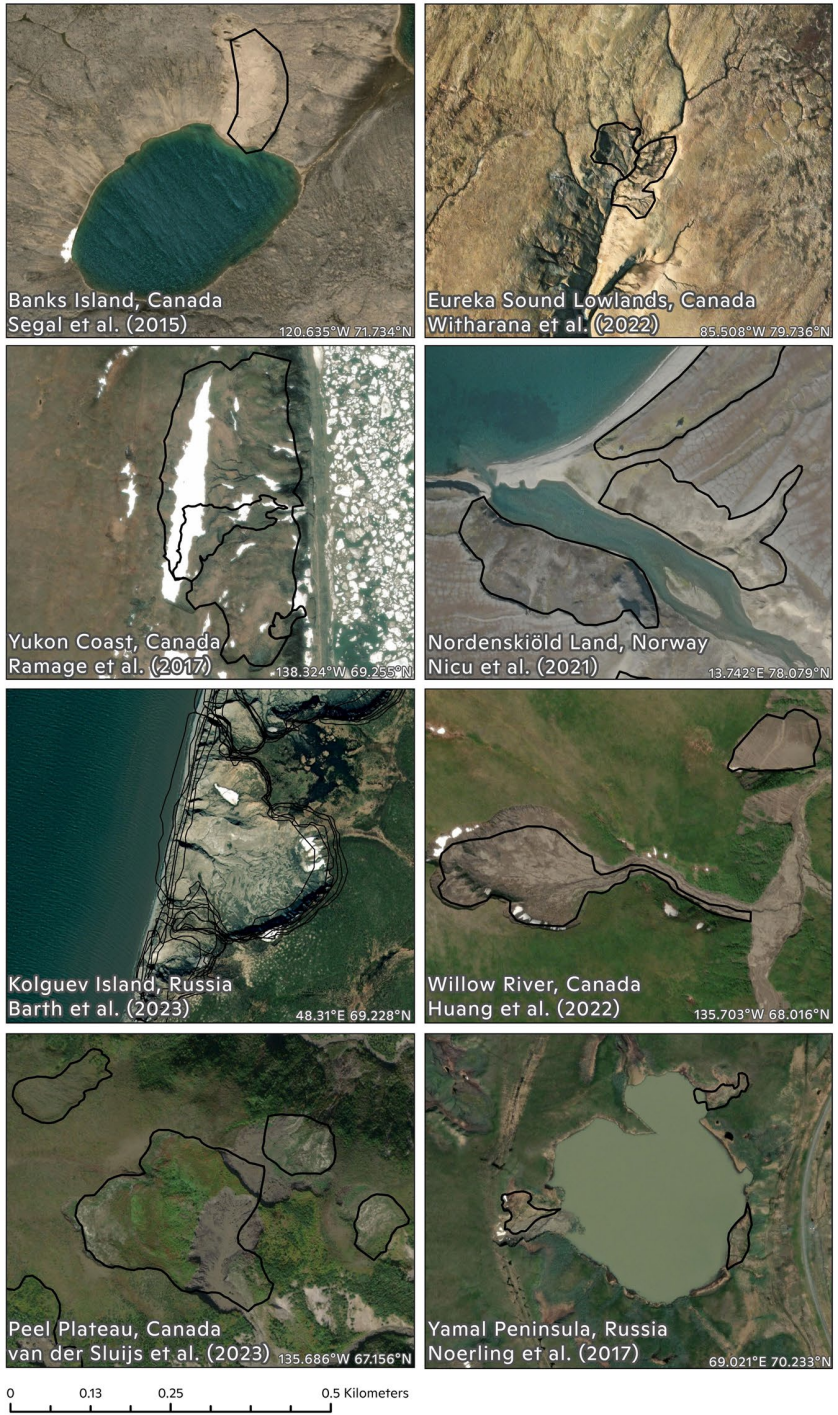
Selected examples of digitised RTS features from the source data sets. The underlying base map is Maxar 0.5 m high-resolution satellite imagery. This shows a range of RTS types, appearances and development stages from various locations across the Arctic.

All raw data sets are associated with at least one peer-reviewed publication except one in preparation (Elia *et al*., in prep.). Beyond the data quality assurance by publications, we also actively verified the data quality by partially inspecting each raw data set. Randomly sampled RTS polygons in the data sets were manually examined to our best ability to verify the genuineness based on a 4 m Maxar base map and a 0.5 m Esri base map. Repeat polygons drawn on the same RTS instance were preserved for use in time-series analyses. We are aware that due to the fast-developing nature of RTS and the different image acquisition times of base maps one can access, there are unavoidable discrepancies between polygons and base maps when used in different studies (Fig. 3). We therefore did not aim for perfect physical alignment between polygons and the base map, but rather focused on verifying that the RTS feature did exist at some point in time. As a result, we note that the data set is designed to require users’ pre-processing through careful filtering and adaption to ensure the best possible alignment between the data set and specific imagery sources.

### Data bias

We acknowledge that the ARTS database incorporates data from various research groups, inevitably introducing differences in visual interpretations^46^. This diversity is crucial for supporting future automated mapping procedures aimed at consistent and multi-temporal RTS mapping. However, users should be aware that potential biases from individual inventories could influence automated mapping models. For example, if a particular group mapped RTSs at specific spatial scales, a complex deep learning algorithm might become sensitive to this preference, potentially affecting results. Similar considerations apply to RTS shape preferences and other morphometric characteristics. We believe the breadth of our global database should help mitigate individual biases with minimal bias-removal techniques required. Nevertheless, we encourage future users to consider bias removal strategies in their models or at least assess their necessity when utilising the ARTS database.

### Data quality check

RTS polygons in the data sets were examined to the best ability to verify the genuineness based on a 4 m Maxar base map and a 0.5 m Esri base map. Repeat polygons drawn on the same RTS instance were preserved for use in time-series analyses. There are some repetitions of RTS delineations due to different groups working in the same area, e.g. Banks Island, in addition to repetitions due to RTS development through time and repeat delineations on images acquired on different dates. We are aware that due to the fast-developing nature of RTS and the different image acquisition times of base maps one can access, there are unavoidable discrepancies between polygons and base maps when used in different studies (Fig. 3). We therefore did not aim for perfect physical alignment between polygons and the base map, but rather focused on verifying that the RTS feature did exist at some point in time. As a result, we note that the data set is designed to require users’ pre-processing through careful filtering and adaption to ensure the best possible alignment between the data set and specific imagery sources.

Due to the cooperative nature of the ARTS data set, the consistency of the RTS digitisation criteria across (and even within) contributors is likely heterogeneous. Therefore, for studies that are highly sensitive to digitisation quality, we recommend a thorough data quality check, filtering, or adaptation before data processing. Second, due to the fast-developing nature of RTS, we recommend a walkthrough of RTS digitisations in use with a base map underlay to check for recent RTS developments that affect the accuracy of the digitisation. Likely, negative data may also be affected by future RTS development and therefore requires verification before use with more recent imagery.

### Generating training-validation-testing patches for DL models

We observed from the data set that the occurrence of RTS features can be discrete, but more frequently they tend to be clustered or adjacent. This nature of RTS leads to a caveat that often more than one RTS can appear in a single training image patch. Therefore, when using the data set in DL model training, a random training-validation-testing split is often problematic. Two or more adjacent RTS can be randomly assigned to the training and testing set while their distance is so close that the testing RTS has already appeared in the training RTS patch, causing data leakage and overestimated model accuracy. Some possible solutions to this issue could be 1) manually selecting the validation and testing set and making sure no training RTS appears in the same scene; 2) using algorithms to ensure that RTS within a certain distance of other RTS are included in the same training/testing set; or 3) using region-based cross-validation. We have developed an automatic data set splitting algorithm which will guarantee completely separated training-validation-testing subsets generated for a given ratio of split. The algorithm is implemented in our ARTS GitHub repository with a tutorial and use instructions. We recommend using this tool to do data-splitting for DL model training.

### Contribution Guideline

The database is open to high-quality contributions regardless of size. There are four or five steps to contribution depending on whether or not new metadata columns are being added:

1. Generate UIDs for all RTS digitisation entries (Automated, see section Metadata Formatting)
2. Check polygon intersection (Automated)
3. Update UIDs using the intersection information (Automated)
4. Request data contribution via GitHub (recommended) or share directly with the correspondence author.

For a data set that has new columns that do not exist in the latest version of the Metadata Format Summary (https://github.com/whrc/ARTS/blob/main/Metadata%20Format%20Summary.csv):

5. Add a new metadata entry type to the Metadata Format Summary, including FieldName, Format and Description.

#### Data Versioning

For a cooperative and evolving data set, it is important to track changes over time and avoid overwriting accidents. Therefore we defined a data versioning scheme to help users understand the data set evolution and allow rolling back to previous versions. We adopted a three-part semantic version number convention consisting of three numbers connected by dots (e.g. 3.0.2). Where the first number indicates the incorporation of a new set of RTS entries from a new data source. The second number indicates batch changes or additions to the metadata (new rows or new columns) without introducing a new data source. The last number indicates minor changes or fixes to the existing data or metadata, such as editing existing metadata or adjusting polygons’ vertices to the existing data. Each version update will require a concise update note describing the changes to the data set.

## Data Availability

Code described in this manuscript can be accessed at the GitHub repository^47^ (https://github.com/whrc/ARTS).
